# High level of serum complement 3 is a risk factor for vascular stenosis progression in TA patients receiving tocilizumab: a prospective observational study

**DOI:** 10.1186/s13075-023-03106-7

**Published:** 2023-08-02

**Authors:** Chen Rongyi, Dai Xiaojuan, Wang Jinghua, Ma Lingying, Dai Xiaomin, Ma Lili, Chen Huiyong, Jiang Lindi, Sun Ying

**Affiliations:** 1 Department of Rheumatology, Zhongshan Hospital Fudan University, No.180, Fenglin Road, Xuhui District, Shanghai, 200032 China; 2 Evidence-Based Medicine Centre, Fudan University, Shanghai, China

**Keywords:** Takayasu arteritis, Tocilizumab, Vascular stenosis progression, Complement 3

## Abstract

**Background:**

The IL-6R antibody tocilizumab has been proven effective in treating Takayasu arteritis (TA). However, some patients show silent vascular stenosis progression (VSP) despite treatment with tocilizumab. The aim of the study was to explore the related risk factors of VSP in patients treated with tocilizumab.

**Methods:**

Patients receiving tocilizumab were enrolled from the prospective living ongoing East China Takayasu Arteritis cohort. Their medical information was uniformly recorded with a homogenized evaluation method. Magnetic resonant angiography or computed tomographic angiography was employed to monitor VSP during the follow-up period, and Cox regression analysis was performed to explore the related risk factors.

**Results:**

Thirty-eight patients were enrolled, among whom 18 (47.4%) experienced VSP, and seven and three patients experienced new and worsened vascular ischemic symptoms and events (VISE) during follow-up, respectively. The median period for VSP occurrence was 6.9 months during follow-up. Patients with VSP showed higher levels of baseline complement 3 (C3) than those in the patients without VSP. Multivariate Cox regression analysis revealed baseline C3 level (hazard ratio [HR] = 7.05, 95% confidence interval: 1.50–33.07, *p* = 0.013) was independently associated with VSP, with a cut-off value of 1.22 g/L.

**Conclusions:**

47.4% of TA patients treated with tocilizumab would suffer VSP. A high C3 level is a risk factor for VSP in TA patients receiving tocilizumab, which may facilitate the option of tocilizumab in the future.

**Supplementary Information:**

The online version contains supplementary material available at 10.1186/s13075-023-03106-7.

## Background

Takayasu arteritis (TA) is a rare type of non-specific inflammatory granulomatous vasculitis that involves the aorta and its main branches [1]. The disease is characterized by the damage of the regional vascular wall structure and function because of inflammation and subsequent pathological repair, manifesting as thickening, stenosis, and vascular dilation [2]. These pathological processes result in the ischemia of the target organ, or rupture of damaged vessels, causing hemorrhage or infarction, seriously affecting the health and quality of life of patients with TA. Therefore, successful inflammation control is at the core of TA treatment.

The combination of glucocorticoids (GCs) and immunosuppressants is the first-line treatment option for TA. The former interrupt the inflammation cascade quickly while the latter result in long-term immunosuppression during GC decrement, reducing the risk of disease relapse. Furthermore, biological agents are recommended for patients with severe or refractory disease [3]. Tocilizumab, a commonly used monoclonal antibody that targets interleukin-6 receptor (IL-6R), has been shown to be effective in reducing inflammation and controlling disease activity in TA in multiple studies, with remission and relapse rates of 74% and 40.6%, respectively, at 6 months as well as significantly decreased GCs [4–6]. However, detailed assessments of angiographic change following tocilizumab treatment are rare.

Vascular stenosis, characterized by lesioned vessels and which is usually evaluated using non-invasive whole-body magnetic resonance angiography (MRA) or computed tomography angiography (CTA), is the most common clinical manifestation of TA [7, 8]. Severe vascular stenoses may lead to the infarction and malfunction of important organs, such as the heart, brain, and kidney, and cause corresponding vascular ischemic symptoms and events (VISE). Although tocilizumab resolves the inflammation and alleviates the vascular stenosis in patients with TA [4, 5, 9], some patients still develop silent vascular stenosis progression (VSP) during the follow-up period [5, 10–13]. However, the characteristics of these TA patients with VSP and the risk factors of VSP remain unclear. Thus, the present study was aimed at investigating VSP in TA patients receiving tocilizumab and exploring the risk factors related to VSP. The results might provide suggestions for selecting tocilizumab therapy to acquire better outcomes in TA patients.

## Materials and methods

### Study design

This prospective observational study was based on the prospective living ongoing East China Takayasu Arteritis (clinical trial no.: NCT03893136) cohort, which includes TA patients who met the classification criteria of the American College of Rheumatology 1990 [14]. All the patients were treated per the designed protocols. Patients were followed-up per the pre-designed plan and evaluated by a specific multidisciplinary expert team. The demographic information and clinical materials, assessed by a special expert team, were collected and summarized in the REDCap database by a specific assistant. Laboratory examinations for the present study including erythrocyte sedimentation rate (ESR), C-reactive protein (CRP), complement 3 (C3), interleukin-6 (IL-6), and serum amylase A (SAA), etc., were performed in the central laboratory in our hospital according to the protocol. The patients underwent MRA or CTA at baseline, every 6 months in the first year, and every year thereafter in general. The vascular types were determined per the 1996 Numano classification for TA by using vascular images [15]. These images were assessed by rheumatologists, radiologists, and vascular surgeons. The study complies with the Declaration of Helsinki. Written informed consent was obtained from all the patients prior to the enrolment, and the study protocol was approved by the ethics committee of Zhongshan Hospital Fudan University (approval no.: B2016-168).

### Patients

In the present study, patients who received tocilizumab therapy in our center were consequently enrolled from the cohort from January 2010 to June 2021. The patient inclusion criteria for the present study were as follows: (1) patients’ condition was complicated by severe or refractory TA; (2) patients with active disease; (3) patients underwent angiographic evaluation and were prescribed tocilizumab consecutively at the time of the administration of the first dose in our center; (4) patients completed the medication course and at least 6 months of follow-up per protocol; (5) patients received consistent treatment consisting of GCs with or without immunosuppressants; and (6) patients were not complicated with chronic infections such as tuberculosis or hepatitis B virus infection. Severe disease was defined as TA with organ- or life-threatening manifestations such as vision loss, syncope, cerebrovascular ischemia, cardiac ischemia, limb ischemia, and kidney ischemia. Refractory disease was defined as persistent active disease consistent with the TA management guidelines put forth by the American College of Rheumatology/Vasculitis Foundation in 2021 despite proper immunosuppressive therapy [16]. Disease activity was evaluated by the NIH score, whereby a score of ≥ 2 was considered indicative of an active phase [17].

### Intervention protocols

All the enrolled patients were prescribed GCs and tocilizumab, as well as immunosuppressants when necessary. Regarding tocilizumab, a dose of 8 mg/kg intravenous administration per month was used regularly. The initial GC dose was 1.0 mg/kg day for treatment-naïve patients in the active phase of the disease [3, 18], while that for previously treated patients could be increased or maintained properly according to the disease status at enrolment, both of which were tapered gradually with the improvement of disease activity according to the NIH score. Immunosuppressants were selected by rheumatologists. They consisted of methotrexate (10 mg per week), leflunomide (10 mg twice per day), rapamycin (1 mg per day), thalidomide (50 mg per night), azathioprine (50 mg per day), and mycophenolate mofetil (0.5 g twice per day).

### Treatment effect

Complete remission at 6 months was defined as follows: (i) no new/worsened systemic symptoms, (ii) no new/worsened vascular symptoms or signs, (iii) GCs dose of ≤ 15 mg/day, and (iv) normal ESR (≤ 40 mm/h) at 6 months. Partial remission was recorded if item (ii) was satisfied combined with any two of items (i), (iii), and (iv). Relapse was defined as the occurrence of a new active disease after complete remission.

### Outcomes

Follow-up was terminated at the first occurrence of VSP or on May 31, 2022. The primary endpoint of the study was the occurrence of VSP. VSP was defined as a decrease in the vascular lumen diameter of more than 20% or the development of new lesions of stenosis in the follow-up imaging during the study period, compared with that in the baseline imaging. The secondary endpoint was the occurrence of VISE, defined as the new or worsened vascular ischemic symptoms and events during follow-up caused by vascular stenosis, validated by objective laboratory or imaging examinations. For example, ischemic stroke was defined as cerebral infarction symptoms and signs, with corresponding ischemic or infarcted photographic changes in the brain MRI or CT imaging. Vision loss was defined as the repeated occurrence of or progressively exacerbated visual abnormalities, validated by the abnormality of imaging exams such as Fundus photography. Furthermore, the relationship between VSP and VISE was explored.

### Statistical analysis

Continuous variables are expressed as mean ± standard error or median (interquartile range, IQR) and were compared with a *t*-test or Wilcoxon rank-sum test according to their normality. Categorical variables are expressed as frequency (percentages) and were compared with the *χ*^2^-test or Fisher’s exact test (sample number =  < 5). Cox regression analysis was performed to explore the potential risk factors of the target, among which indices with a *p*-value of < 0.1 were included in a further multivariate Cox regression analysis, the results of which were further analyzed by plotting Kaplan–Meier survival curves. Time-dependent receiver operating characteristic (ROC) curves were plotted to identify the optimal cut-off point and the value of related risk factors to predict VSP during the follow-up period. A *p*-value of < 0.05 in the two-sided test was considered statistically significant. Statistical analyses were performed with the SPSS 26.0 and R software.

## Results

### Overall baseline characteristics of the patients

In all, 38 patients with TA were enrolled in the present study, among whom 29 (76.3%) had severe disease. The percentage of female patients was 92.1% (35 cases), and the age of the enrolled patients was 27.3 ± 10.7 years. The median disease duration was 12.9 months; 47.3% of the cases were treatment naïve. The most common vascular types were type I (26.3%) and type V (50%). The most common symptom was fatigue (21.1%). Moreover, 28.9% and 34.2% of cases showed signs of vascular murmurs and pulselessness, respectively. Inflammatory markers including ESR, CRP, C3, IL-6, and SAA increased significantly compared with the corresponding normal upper limit reference value (Table 1, Supplementary Table S1).

**Table 1:** The baseline demographic characteristics of the patients with and without VSP during follow-up

	**Total** **(*n* = 38)**	**Non-VSP** **(*n* = 20)**	**VSP** **(*n* = 18)**	***p-*value**
Age, years	27.3 ± 10.7	30.8 ± 11.8	23.6 ± 8.4	0.040
Gender (female), *n* (%)	35 (92.1)	19 (95.0)	16 (88.9)	0.595
Disease duration, months	12.9 (5.0–33.4)	16.9 (6.4–94.1)	9.0 (5.0–15.3)	0.151
VSP period, months	7.0 (5.8–10.8)	7.3 (6.0–12.6)	6.9 (5.6–9.2)	0.745
Naïve, *n* (%)	18 (47.3)	8 (40.0)	10 (55.6)	0.338
Refractory/severe, *n* (%)	9 (23.7)/29 (76.3)	4 (20.0)/16 (80.0)	5 (27.8)/13 (72.2)	0.709
Hypertension, *n* (%)	5 (13.2)	4 (20.0)	1 (5.6)	0.344
**Vascular types, *n* (%)**				0.389
I	10 (26.3)	5 (25.0)	5 (27.8)	
IIa	4 (10.5)	2 (10.0)	2 (11.1)	
IIb	5 (13.2)	1 (5.0)	4 (22.2)	
V	19 (50)	12 (60.0)	7 (38.9)	
**Symptoms**				
Fever, *n* (%)	4 (10.5)	2 (10)	2 (11.1)	1.000
Amaurosis, *n* (%)	4 (10.5)	3 (15)	1 (5.6)	0.606
Neck pain, *n* (%)	5 (13.2)	3 (15)	2 (11.1)	1.000
Visual loss, *n* (%)	5 (13.2)	3 (15)	2 (11.1)	1.000
Thoracalgia, *n* (%)	6 (15.8)	3 (15)	3 (16.7)	1.000
Fatigue, *n* (%)	8 (21.1)	4 (20)	4 (22.2)	1.000
**Signs**				
Pulselessness, *n* (%)	11 (28.9)	5 (25)	6 (35.3)	0.495
Vascular murmur, *n* (%)	13 (34.2)	3 (15)	10 (58.8)	0.008
**Laboratory parameters**				
Hemoglobin, g/L	112.3 ± 18.6	112.1 ± 23.0	112.6 ± 12.6	0.933
WBC, × 10^9^/L	8.9 ± 3.5	8.4 ± 3.8	9.5 ± 3.2	0.371
Platelet, × 10^9^/L	329.4 ± 108.9	294.4 ± 95.2	368.6 ± 112.4	0.039
ALT, U/L	11.0 (8.0–19.0)	12.4 ± 6.7	15.6 ± 9.9	0.255
Albumin, g/L	42.0 (39.0–43.0)	42.0 (39.0–46.0)	42.0 (40.3–43.0)	0.533
Globin, g/L	29.29 ± 4.90	27.53 ± 4.48	31.38 ± 4.67	0.018
Creatinine, μmol/L	54.0 (47.0–67.0)	72.2 ± 52.9	53.9 ± 14.9	0.190
Blood urine nitrogen, μmol/L	4.8 ± 1.7	4.8 ± 1.8	4.7 ± 1.5	0.816
**Inflammatory markers**				
ESR, mm/h	44.8 ± 30.6	35.8 ± 26.1	54.9 ± 32.7	0.053
CRP, g/L	12.4 (1.6–39.5)	17.3 ± 23.5	37.8 ± 41.7	0.069
C3, g/L	1.20 ± 0.28	1.07 ± 0.19	1.34 ± 0.30	0.005
C4, g/L	0.26 ± 0.08	0.25 (0.20–0.27)	0.27 (0.22–0.34)	0.240
CH50, g/L	68.0 ± 21.5	61.8 ± 20.5	74.6 ± 21.2	0.100
IgG, g/L	12.9 ± 3.3	12.4 ± 3.0	13.4 ± 3.6	0.382
IL-6, pg/ml	9.3 (3.9–18.5)	7.2 (3.7–13.6)	14.4 (5.4–26.8)	0.156
IL-8, pg/ml	7.3 (5.0–9.2)	7.0 (5.0–9.0)	8.0 (5.0–22.0)	0.493
SAA, mg/L	30.4 (5.7–175.8)	9.1 (5.1–118.0)	104.8 (21.8–289.8)	0.023
NIH score	3.0 (2.8–4.0)	3.0 (2.0–3.8)	3.0 (3.0–4.0)	0.105
**Concomitant Immunosuppressants types, *n* (%)**				0.194
0	23 (60.5)	12 (60.0)	11 (61.1)	
1	12 (31.6)	5 (25.0)	7 (38.9)	
2	3 (7.9)	3 (15.0)	0	

The immunosuppressants include methotrexate (10 cases), LEF (3 cases), rapamycin (3 cases), thalidomide (1 case), azathioprine (1 case), and mycophenolate mofetil (1 case)

Reference values: ESR, < 20 mm/h; CRP, < 3 mg/L; C3, 0.70–1.40 g/L; C4, 0.10–0.40 g/L; CH50, 50.0–100.0 g/L; IgG, 8.60–17.40 g/L; IgA, 1.00–4.20 g/L; IgE, < 200 IU/ml; IL-6, < 3.4 pg/ml; IL-8, < 62 pg/ml; SAA, 0–6.4 mg/L

*Abbreviations*: *VSP *vascular stenosis progression during follow-up, *WBC *white blood cells, *ALT *alanine transaminase, *BUN *blood urine nitrogen, *ESR *erythrocyte sedimentation rate, *CRP *C-reactive protein, *C3 *complement 3, *C4 *complement 4, *CH50 *50% hemolytic complement, *IgG *immunoglobin G, *IgA *immunoglobin A, *IgE *immunoglobin E, *IL-6 *interleukin-6, *IL-8 *interleukin-8, *SAA *serum amyloid A, *NIH score *National Institutes of Health score

Continuous variables are expressed as mean ± standard error or median (interquartile range, IQR) according to their normality

### Medication

During the follow-up period, all patients were treated with GCs and tocilizumab. The initial GC dose was 0.67 ± 0.30 mg/kg day. Moreover, 31.6% and 7.9% of cases were prescribed extra one and two kinds of immunosuppressants simultaneously, respectively. The most common immunosuppressant was methotrexate (10 cases, 26.3%) (Table 2). The median period of tocilizumab use was 9.36 (6.94–15.91) months, and the longest period of use was 26 months (Supplementary Table S1).

**Table 2:** Dynamic changes in prednisone concentrations and events occurring during the follow-up period

	**Total** **(*n* = 38)**	**Non-VSP** **(*n* = 20)**	**VSP** **(*n* = 18)**	***p-*value**
**Prednisone**				
Baseline, mg/kg day	0.67 ± 0.30	0.61 ± 0.34	0.74 ± 0.23	0.233
Follow-up at 6 months, mg/kg day	0.28 (0.21–0.35)	0.25 (0.21–0.33)	0.29 (0.23–0.45)	0.449
Follow-up at 12 months, mg/kg day	0.14 (0.10–0.22)	0.14 (0.10–0.18)	0.14 (0.12–0.22)	0.667
**VISE**	10 (26.3)	2 (10.0)	8 (44.4)	0.027
Ischemic stroke	4 (10.5)	1 (5.0)	3 (16.7)	
Visual loss	4 (10.5)	1 (5.0)	3 (16.7)	
Amaurosis	2 (5.3)	0	2 (11.1)	
Angina	1 (2.6)	0	1 (5.6)	
Heart failure	1 (2.6)	1 (5.0)	0	
Intermittent claudication	1 (2.6)	0	1 (5.6)	
**Treatment effect**				
Complete remission	20 (52.6)	10 (50.0)	10 (55.6)	0.732
Partial remission	11 (28.9)	6 (54.5)	5 (45.5)	0.880
Relapse	2 (16.7)	0	2 (40.0)	0.152
**Recorded infection**				0.218
Respiratory system infection	2 (5.3)	0	2 (11.1)	
Digestive system infection	1 (2.6)	0	1 (5.6)	
Reproductive system infection	1 (2.6)	0	1 (5.6)	
Skin infection	1 (2.6)	1 (5.0)	0	
Blood^a^	1 (2.6)	0	1 (5.6)	
**VSP locations**				
Subclavian artery			16 (42.1)	
Carotid artery			2 (5.3)	
Brachiocephalic trunk			2 (5.3)	
Renal artery			1 (0.03)	
Iliac artery			1 (0.03)	

^a^The patient was diagnosed with hematogenous infection by T-SPOT and NGS-sequence in peripheral blood

### Treatment effects

**Fig. 1:**
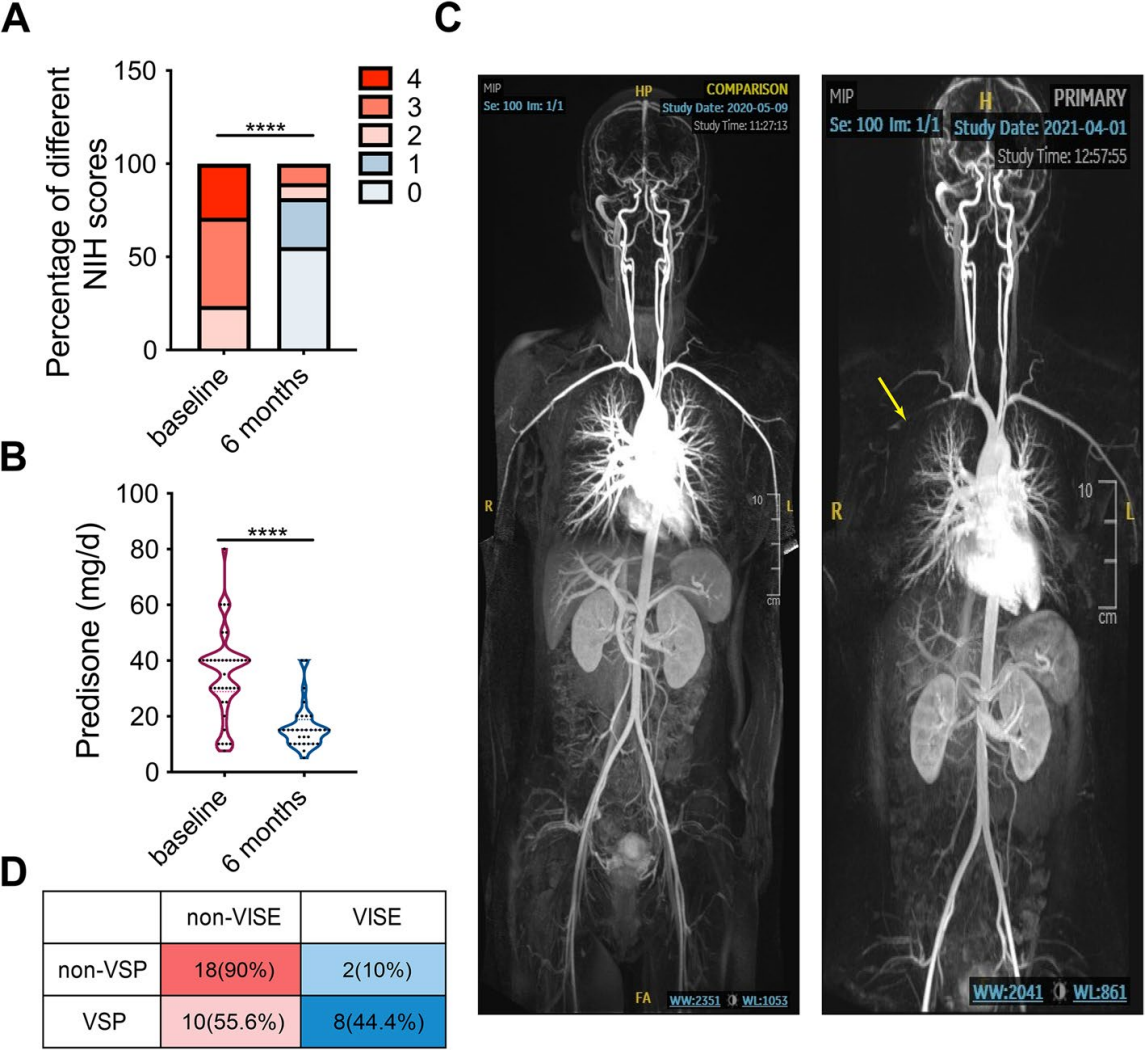
The treatment effect of tocilizumab. **A** The NIH scores of enrolled patients at baseline and after 6 months of treatment. **B** The dose of prednisone in enrolled patients at baseline and positive treatment effect of tocilizumab. **C** representative image of VSP in TA. **D** The incidence of VSP and VISE during follow-up. ****p < 0.0001. TA, Takayasu arteritis; VSP, vascular stenosis progression; VISE, vascular ischemic symptoms and events

**Fig. 2:**
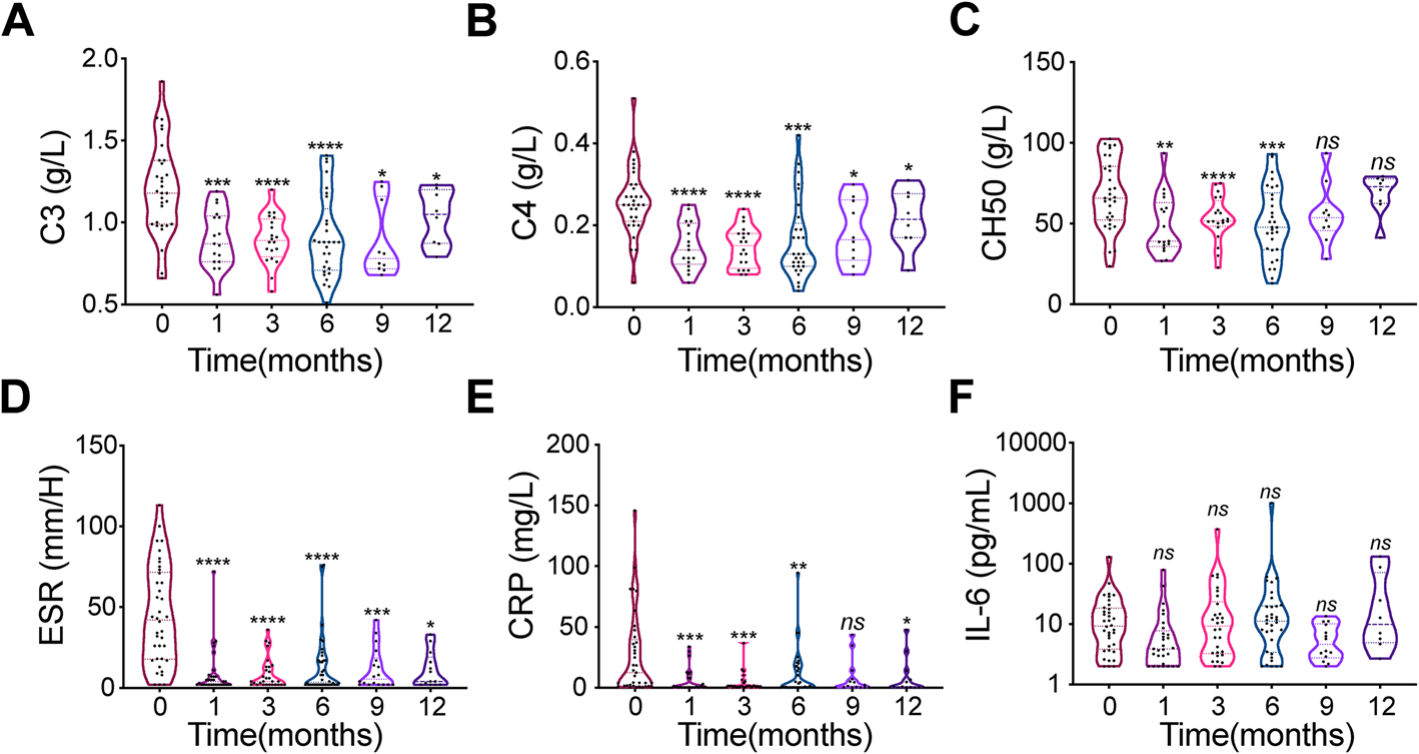
Dynamic changes in inflammatory markers during tocilizumab use in TA patients. **A** Dynamic changes in C3, C4, and CH50 in the 12 months of follow-up. **B** Dynamic changes in ESR, CRP, and IL-6 in the 12 months of follow-up. *Compared with baseline (0 month); *p < 0.05; **p < 0.01; ***p < 0.001; ****p < 0.0001. C3, complement 3; C4, complement 4; CH50, 50% hemolytic complement; ESR, erythrocyte sedimentation rate; CRP, C-reactive protein; IL-6, interleukin-6

At 6 months, the overall remission rate was 86.1% (31 in 36 cases), with the complete and partial remission rates being 30.6% (11 cases) and 50.6% (20 cases), respectively. The relapse rate at 12 months was 16.7% (two in 12 cases) (Table 2). The disease activity NIH score significantly decreased after treatment for 6 months. The median GC doses after 6 and 12 months of treatment were 0.28 and 0.14 mg/kg day, respectively. The GC dose and inflammatory markers including ESR, and levels of CRP, C3, complement 4 (C4), and 50% hemolytic complement (CH50) decreased significantly compared with the corresponding baseline values (Figs. 1A, B and 2). Six cases of infection, including those of the respiratory, digestive, urinary, reproductive systems, and blood were recorded (Table 2).

### VSP during the follow-up period

Overall, VSP was noted in 18 cases (47.3%) during the follow-up period; the follow-up period was 6.9 (range: 5.6–9.2) months. The subclavian artery (42.1%), brachiocephalic trunk (5.3%), and carotid artery (5.3%) were the most vulnerable in terms of lesion development. The VSP was manifested as aggravated vascular stenosis in preexisting narrow vessels mainly, among which no new lesions were observed in these vessels (Table 2, Fig. 1C). At baseline, there was a history of VISE in 28 cases (73.7%). During the follow-up period, 10 patients (26.3%) experienced VISE, with new VISE in seven cases and worsened VISE in three cases. The most common VISE among these were ischemic stroke (10.5%) and vision loss (7.9%). Further analysis revealed that patients with VSP had a significantly increased incidence of VISE during the follow-up period (44.4% vs 10%, *p* = 0.027) (Table 2, Fig. 1D, Supplementary Table S1, Supplementary Figure S1).

### Characteristics of patients with and without VSP at baseline and during the follow-up period

At baseline, compared with patients without VSP, those with VSP were much younger (age: 30.8 ± 11.8 years vs 23.6 ± 8.4, *p* < 0.040) and had a higher incidence of vascular murmurs (15% vs 58.8%, *p* = 0.008). There were no significant intergroup differences in sex, disease duration, follow-up period, percentage of treatment-naïve patients, vascular types, and symptoms. Moreover, the status of hypertension was not significantly different as well. However, compared with patients without VSP, those with VSP much a higher platelet count (294.4 ± 95.2 vs 368.6 ± 112.4 × 10^9^/L, *p* < 0.039), and levels of globin (27.53 ± 4.48 vs 31.38 ± 4.67 g/L, *p* = 0.018), C3 (1.07 ± 0.19 vs 1.34 ± 0.30 g/L, *p* = 0.005), and SAA (9.1 [5.1–118.0] vs 104.8 [21.8–289.8] mg/L, *p* = 0.023) at baseline, although the levels of ESR, CRP, and IL-6 were not significantly different between the two groups (Table 1). During the follow-up period, there were no significant intergroup differences in GC dose; immunosuppressant types; changes in inflammatory markers including levels of C3, ESR, CRP, and IL-6; and complete remission, partial remission, and relapse rates after 6 months of treatment (Table 2, Supplementary Figure S2).

However, patients with VISE had higher levels of C3 (1.37 ± 0.27 vs 1.14 ± 0.26, *p* = 0.033). Patients who did and did not experience VISE showed similar symptoms; laboratory parameters including hemoglobin and creatinine; inflammatory markers including ESR, CRP, and IL-6 at baseline; and the remission rate. Further analysis revealed that these parameters were also not significantly different in patients showing VSP who did and did not experience VISE (Supplementary Tables S2 and S3).

### Risk factor analysis for VSP and survival analysis

**Fig. 3:**
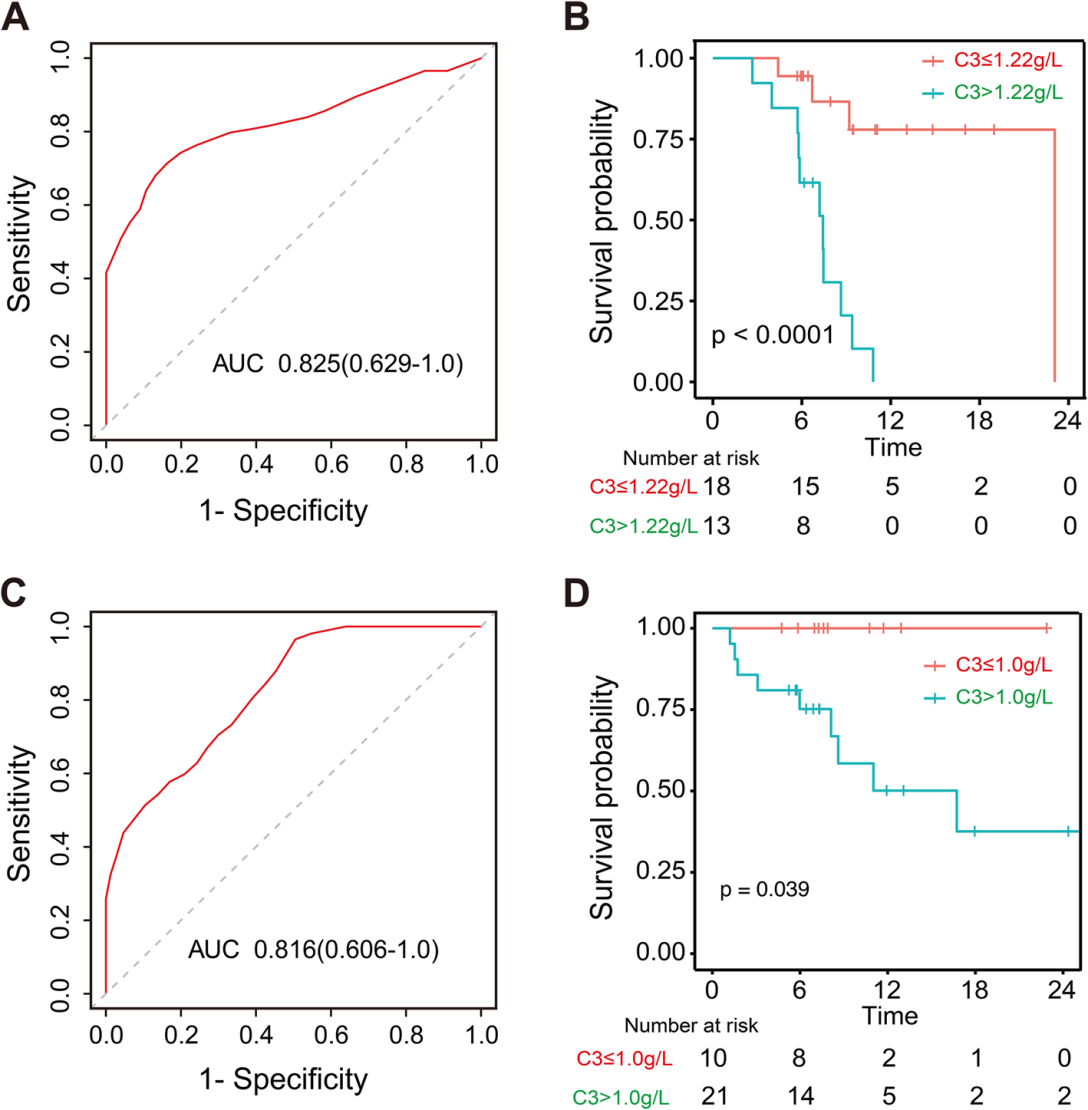
Value of C3 in predicting VSP treated with tocilizumab in patients with TA. **A** ROC curves of C3 for identifying VSP in 1 year (cut-off level: 1.22 g/L). **B** Kaplan-Meir curves of VSP with respect to different C3 levels. **C** ROC curves of C3 for identifying VISE in 1 year (cut-off level: 1.0 g/L). **D** Kaplan-Meir curves of VISE with respect to different C3 levels. C3, complement 3; ROC, receiving operating characteristic; VSP, vascular stenosis progression; VISE, vascular ischemic symptoms and events

The univariate Cox regression analysis showed that age (HR: 0.95, 95% confidence interval [CI]: 0.90–1.01, *p* = 0.090), levels of globin (HR: 1.17, 95% CI: 1.04–1.31, *p* = 0.012), and C3 (HR: 11.68, 95% CI: 1.86–73.4, *p* = 0.003), together with ESR, CRP, and IL-6 at baseline were associated with VSP but not treatment-naïve, hypertension statuses, or the immunosuppressant used. The multivariate Cox regression analysis showed that C3 level (HR: 7.05, 95% CI: 1.50–33.07, *p* = 0.013) was independently associated with VSP after adjustment for age and levels of globin, IL-6, ESR, and CRP (Table 3). Time-dependent ROC curve analysis showed that the cut-off C3 level for identifying VSP in 1 year was 1.22 g/L (area under the curve, AUC: 0.825) (Fig. 3A, C).

**Table 3:** Factors associated with VSP in patients treated with tocilizumab

	**Univariate Cox regression analysis**		**Multivariate Cox regression analysis**	
	HR (95% CI)	*p-*value	HR (95% CI)	*p*-value
Age	0.95 (0.90–1.01)	0.090	0.95 (0.88–1.03)	0.230
Sex	2.00 (0.45–8.91)	0.363		
Disease duration	0.97 (0.92–1.02)	0.205		
Naïve	1.64 (0.62–4.30)	0.316		
Hypertension	0.83 (0.11–6.32)	0.856		
Pulselessness	1.07 (0.38–2.96)	0.902		
Vascular murmurs	1.89 (0.70–5.13)	0.212		
Globin	1.17 (1.04–1.31)	0.012	1.04 (0.86–1.26)	0.667
ESR	1.02 (1.01–1.04)	0.005	1.00 (0.96–1.04)	0.965
CRP	1.02 (1.01–1.03)	0.006	1.01 (0.97–1.05)	0.588
IL-6	1.02 (1.00–1.04)	0.055	1.00 (0.96–1.05)	0.910
IL-8	1.02 (0.98–1.05)	0.412		
C3	11.68 (1.86–73.4)	0.003		
C3 > 1.22 g/L	9.17 (2.48–34.00)	0.001	7.05 (1.50–33.07)	0.013
C4	7.35 (0.04–1326.80)	0.452		
NIH score	1.50 (0.77–2.91)	0.235		

*Abbreviations*: *ESR *erythrocyte sedimentation rate, *CRP *C-reactive protein, *C3 *complement 3, *C4 *complement 4, *CH50 *50% chemolytic complement, *IL-6 *interleukin-6, *IL-8 *interleukin-8, *NIH score *National Institutes of Health score

Moreover, C3 levels were significantly related to VISE during the follow-up period (HR: 14.17, 95% CI: 1.39–143.94, *p* = 0.025), despite the distribution of C3 being similar in patients who did and did not experience VISE at baseline. The cut-off C3 level for the identification of VISE was 1.0 g/L (AUC: 0.816) (Supplementary Table S4). Further survival analysis revealed that the incidences of VSP and VISE during the follow-up period were much higher in patients with higher levels of C3 at baseline, rather than different NIH scores (Fig. 3B, D, Supplementary Figure S3).

## Discussion

This prospective observational study focuses on VSP in TA patients treated with tocilizumab. Although tocilizumab was effective in alleviating inflammation and disease activity, approximately 47.3% of TA patients receiving tocilizumab showed VSP during the follow-up period. This finding was consistent with those of previous reports, which did not attract enough attention (Summarized in Supplementary Table S5). Furthermore, C3 was a predictive factor for VSP, with an AUC of 0.825 in 1 year. We believe our findings would inform tocilizumab prescription in TA patients.

Tocilizumab was an effective drug and has been widely used in TA management. Both double-blind randomized placebo-controlled studies and multicenter retrospective studies have shown the efficiency of tocilizumab in treating TA, especially in cases of severe or refractory disease. Tocilizumab alleviated symptoms, facilitated the reduction of GC dose, decreased the levels of inflammatory markers, and reduced the TA relapse rate with limited safety concerns [5, 6]. Besides, another study showed that the complete remission rate was 70% after 6 months of tocilizumab treatment in 37 TA patients, of whom four developed severe infections, during the follow-up period [19]. Comparison between tocilizumab and cyclophosphamide revealed that tocilizumab was superior in induction treatment [20]. These phenomena are consistent with the results of a decreased GC dose, inflammatory markers, and disease activity score with a total remission rate of 81.5% after tocilizumab treatment in the majority of patients with severe or refractory TA in the present study. Hence, tocilizumab should still be regarded as an important intervention strategy in patients with severe and refractory TA [16]. However, another randomized controlled trial about tocilizumab in refractory TA patients revealed that tocilizumab was not superior to placebo for time to relapse [6], implying that tocilizumab might be not suitable for all TA patients and screening appropriate patients would be an important issue to achieve the best benefit in the next stage.

The inflammatory cytokine IL-6 is involved in the pathogenesis of TA. The risk locus in *IL-6* might increase disease susceptibility by suppressing the expression of the anti-inflammatory gene *GPNMB* [21]. IL-6 could promote vascular fibrosis through the Jak/Stat3 signal pathway targeting aortic adventitial fibroblasts in TA [22, 23], which could be theoretically blocked by the anti-inflammatory IL-6R antibody tocilizumab. Some treated patients showed improvements in vascular lesions [9, 24, 25]. However, VSP was reported with the use of tocilizumab in clinical settings in some cases [5, 10–13]. This finding is consistent with that of the present study, highlighting the complexity and challenge of mechanism exploration and treatment for TA. Therefore, more attention should be paid to this group of patients.

The complement system is tightly associated with TA. C3 was found to be an independent predictive factor for VSP in the present study. This finding was consistent with the previous finding that elevated C3 levels imply an active TA status [26]. In fact, as acute-phase proteins, the levels of complement components including C3 and C4b increase in the serum of TA patients [27]. However, the finding that C3, instead of C4, ESR, or CRP, increased in TA patients with VSP implied that C3 was involved in the pathogenesis from the perspective of molecular mechanisms. Complement-dependent cytotoxicity or the function of downstream complement components might be important reasons underlying this finding [26, 27]. From a clinical setting perspective, more aggressive angiographic evaluation and VISE monitoring during tocilizumab use should be considered in patients with a C3 level higher than 1.22 g/L. However, what should not be neglected for using the proposed biomarker C3 is that the narrow “therapeutic window” in the average levels both between VSP and non-VSP patients, between VISE and non-VISE patients, might increase the misjudgment. More caution should be paid when prescribing tocilizumab according to C3 level, considering the limited sample size of the study. Moreover, further investigations are still warranted to validate the relationship and clarify the exact mechanism between C3 and VSP or VISE.

In the present study, VSP during the follow-up period was noted in 18 cases of severe or refractory TA, with the subclavian arteries being the most vulnerable. Patients with VSP had a higher incidence of VISE, and VISE were consistent with VSP to some extent, indirectly reflecting the causality between VSP and VISE and highlighting the importance and necessity of identifying VSP in a timely manner. VSP together with VISE negatively affected the life quality and resulted in poor prognoses of patients, further emphasizing the importance of identifying the risk factors of VSP and screening this group of TA patients in the early phase.

The study has some limitations as well. First, this was an observational single-arm study with limited sample size and follow-up periods. Moreover, the exact reasons such as inflammation or pathological repair that might account for the study findings remain unclear. Hence, further investigation of potential mechanisms is still warranted.

In conclusion, VSP was not rare (47.4%) in TA patients treated with tocilizumab and GCs. A high C3 level (> 1.22 g/L) is a risk factor for VSP in these patients, and tocilizumab should be used more prudently in this group of TA patients.

## Supplementary Information

**Additional file 1: Supplementary Table S1.** Information of enrolled patients.

**Additional file 2: Supplementary Table S2-4 and Figure S1-3.** Analysis of enrolled patients.

**Additional file 3: Supplementary Table S5.** Summary of published literature on the issue that vascular stenosis progression in TA patients receiving tocilizumab.

## Data Availability

The data is available from the corresponding authors upon reasonable request.

## Abbreviations

AUC: Area under the curve
C3: Complement 3
CH50: 50% Hemolytic complement
CRP: C-reactive protein
ESR: Erythrocyte sedimentation rate
GC: Glucocorticoid
HR: Hazard ratio
IL-6: Interleukin-6
SAA: Serum amylase A
TA: Takayasu arteritis
VISE: Vascular ischemic symptoms and events
VSP: Vascular stenosis progression
