# Supplementary material for: High level of serum complement 3 is a risk factor for vascular stenosis progression in TA patients receiving tocilizumab: a prospective observational study
Source: Arthritis Res Ther. 2023 Aug 2;25:137. doi: 10.1186/s13075-023-03106-7 (PMC10394800; doi:10.1186/s13075-023-03106-7)
Supplement: Supplementary file 1 — Additional file 1: Supplementary Table S1. Information of enrolled patients. [file 13075_2023_3106_MOESM1_ESM.docx]

**Supplementary Table S1. Characteristics of the enrolled patients**

| **No.** | **Age** | **Sex** | **naïve** | **Previous medication** | **Combined IS** | **Reasons** | **VSP location** | **VISE detail** | **VISE period** | **VSP period** | **Toc period** | **Subsequent strategy** |
| --- | --- | --- | --- | --- | --- | --- | --- | --- | --- | --- | --- | --- |
| 1 | 20 | Female |  | Pred |  | Severe | middle and distal part of right subclavian artery |  | 7.29 | 7.20 | 7.29 | Pred +LEF+Rapa |
| 2 | 24 | Female |  | Pred+LEF | LEF | Refractory | left subclavian artery and left common carotid artery |  | 6.90 | 2.66 | 6.90 | Pred+LEF+MMF |
| 3 | 19 | Female |  | Pred+MMF | Rapa | Severe | obvious aggravated vascular stenosis in the right subclavian artery | Cerebrovascular infarction, and visual loss in the right eye, receiving the laser surgery; | 1.74 | 9.40 | 13.24 | Pred +LEF |
| 4 | 32 | Female |  | Pred +tofactinib |  | Refractory | right subclavian artery |  | 5.68 | 5.72 | 5.68 | Pred +MTX |
| 5 | 26 | Female |  | Pred+MTX |  | Severe | right subclavian artery |  | 3.12 | 4.40 | 4.24 |  |
| 6 | 16 | Female | Yes |  | MTX | Severe | double subclavian artery, and auxiliary artery |  | 1.22 | 3.98 | 9.26 | Pred +MTX |
| 7 | 11 | Male | Yes |  | MTX | Severe | right subclavian artery in the more extended area |  | 17.94 | 5.85 | 17.94 |  |
| 8 | 18 | Male | Yes |  |  | Severe | the right subclavian artery |  | 5.78 | 5.78 | 5.78 |  |
| 9 | 19 | Female | Yes |  |  | Severe | the right subclavian artery | stable effort angina | 16.85 | 23.06 | 23.06 |  |
| 10 | 39 | Female | Yes |  |  | Severe | the right internal iliac artery | fainted on the ground with blurred vision; dizziness and fainting, MRI revealed ischemic stroke | 8.18 | 10.81 | 17.15 |  |
| 11 | 44 | Female | Yes |  |  | Severe | right subclavian artery | seizures; Blurred vision, numbness of fingers and sleeve-like sensation | 6.01 | 8.64 | 8.77 |  |
| 12 | 28 | Female | Yes |  |  | Severe | the left carotid artery and the double side subclavian arteries | Acute cerebral infarction of the corpus callosum | 8.67 | 7.46 | 11.76 |  |
| 13 | 28 | Female | Yes |  |  | Severe | the proximal part of renal artery | headache, cough (CT shows hypoperfusion of the brain); renal function decreased | 6.24 | 6.28 | 6.28 |  |
| 14 | 14 | Female | Yes |  |  | Severe | the left thoracic collateral artery, occluded now |  | 15.47 | 5.32 | 17.41 |  |
| 15 | 18 | Female | Yes |  | MTX | Severe | the right subclavian artery |  | 11.70 | 6.70 | 11.70 |  |
| 16 | 25 | Female |  | Pred+CTX | MTX | Refractory | the double sides of subclavian arteries |  | 7.26 | 9.20 | 9.20 | Pred +MTX |
| 17 | 24 | Female |  | Pred+LEF |  | Refractory | the right subclavian artery |  | 7.33 | 7.43 | 7.33 |  |
| 18 | 20 | Female |  | Pred+LEF | MTX | Refractory | right subclavian artery |  | 17.87 | 17.91 | 17.87 |  |
| 19 | 34 | Female |  | Pred+LEF | MTX +Rapa | Refractory |  |  | 11.93 | 6.74 | 11.93 |  |
| 20 | 26 | Female |  | Pred+MTX+Tha | MTX + Tha | Refractory |  |  | 6.41 | 9.46 | 9.46 | Pred+MMF |
| 21 | 26 | Female |  | Pred |  | Severe |  |  | 5.22 | 6.14 | 6.24 |  |
| 22 | 26 | Female |  | Pred + MMF/LEF | LEF + Rapa | Refractory |  |  | 25.89 | 14.82 | 25.89 |  |
| 23 | 40 | Female | Yes |  |  | Severe |  |  | 4.73 | 5.68 | 4.73 | Pred +LEF+Rapa |
| 24 | 22 | Female | Yes |  |  | Severe |  |  | 5.91 | 5.98 | 5.91 | Pred+LEF |
| 25 | 36 | Female |  | Pred+LEF |  | Severe |  |  | 15.84 | 4.11 | 15.84 |  |
| 26 | 19 | Female | Yes |  | MTX | Severe |  |  | 7.89 | 7.92 | 7.89 | Pred + Rapa +LEF |
| 27 | 35 | Female | Yes |  |  | Severe |  |  | 5.85 | 5.91 | 5.85 | Pred+LEF |
| 28 | 13 | Female |  | Pred |  | Severe |  |  | 1.54 | 6.08 | 7.23 | Pred +MTX |
| 29 | 30 | Female |  | Pred+tacrolimus | MTX | Severe |  |  | 24.34 | 18.96 | 24.34 | Pred +MTX |
| 30 | 17 | Female | Yes |  | LEF | Severe |  |  | 7.59 | 6.41 | 7.59 | Pred+LEF |
| 31 | 36 | Female |  | Pred |  | Severe |  |  | 22.87 | 17.02 | 22.87 |  |
| 32 | 32 | Female |  | Pred+AZA+Thal | LEF | Severe |  |  | 7.89 | 4.73 | 7.89 |  |
| 33 | 29 | Female |  | Pred +AZA |  | Refractory |  |  | 16.13 | 16.13 | 16.13 | Stopped (for pregnancy) |
| 34 | 59 | Female | Yes |  |  | Severe |  | progressed renal function decline | 6.97 | 6.01 | 6.97 | stopped (for uncontrolled HBP and ESRD) |
| 35 | 33 | Female | Yes |  |  | Severe |  |  | 10.74 | 10.94 | 10.74 |  |
| 36 | 55 | Female |  | Pred |  | Severe |  | Subacute lacunar infarction in the left frontal lobe, basilar artery aneurysm; heart failure | 11.10 | 11.07 | 11.10 | Pred+tofacitinib; PCI |
| 37 | 14 | Male | Yes |  | MTX | Severe |  |  | 12.91 | 9.43 | 12.91 |  |
| 38 | 33 | Female | Yes | Pred |  | Severe |  |  | 13.08 | 13.08 | 13.08 |  |

Note: 1. Abbreviations: IS, immnosuppresants; VSP, vascular stenosis progress; Pred, glucocorticoid; MTX, methotrexate; LEF, leflunomide; Rapa, rapamycin; MMF, mycophenolate mofetil; AZA, azathioprine; Thal, thalidomide; +, simultaneous prescription; /, use of one immunosuppressant initially followed by switching to another immunosuppressant; VISE, vascular ischemic symptoms and events.

2. VSP period: the date between the first MRA/CTA and the follow-up MRA/CTA observing VSP or the termination. VISE period: the date between the first MRA/CTA performed, and the recorded VISE. Toc period, the date between the first MRA/CTA performed, and the last dose of tocilizumab.

3. All enrolled patients received glucocorticoid prescriptions.

4. The unit of period is month.
