# Supplementary material for: High level of serum complement 3 is a risk factor for vascular stenosis progression in TA patients receiving tocilizumab: a prospective observational study
Source: Arthritis Res Ther. 2023 Aug 2;25:137. doi: 10.1186/s13075-023-03106-7 (PMC10394800; doi:10.1186/s13075-023-03106-7)

**Supplementary Table S2**. The demographic characteristics of patients with and without VISE.

|  | **Non-VISE group**  **(n = 28)** | **VISE group**  **(n = 10)** | ***p-*value** |
| --- | --- | --- | --- |
| Age, years | 26.9±9.9 | 28.7±13.5 | 0.656 |
| Gender (Female), n (%) | 25(89.3) | 10(100) | 0.552 |
| Disease duration, months | 12.9(5.6-48.1) | 10.7(4.7-47.3) | 1.000 |
| VISE period, months | 7.9(6.5-15.7) | 6.1(1.7-9.3) | 0.713 |
| Naïve, n (%) | 12(42.9) | 6(60.0) | 0.468 |
| **Symptoms** |  |  |  |
| Fever, n (%) | 3(10.7) | 1(10.0) | 1.000 |
| Amaurosis, n (%) | 3(10.7) | 1(10.0) | 1.000 |
| Neck pain, n (%) | 4(14.3) | 1(10.0) | 1.000 |
| Visual loss, n (%) | 4(14.3) | 1(10.0) | 1.000 |
| Thoracalgia, n (%) | 5(17.9) | 1(10.0) | 1.000 |
| Fatigue, n (%) | 4(14.3) | 4(40.0) | 0.170 |
| **Signs** |  |  |  |
| Pulseless, n (%) | 7(25.9) | 4(40.0) | 0.442 |
| Vascular murmur, n (%) | 7(25.9) | 6(60.0) | 0.118 |
| **Laboratory parameters** |  |  |  |
| Hemoglobin, g/L | 111.2±20.8 | 115.2±11.0 | 0.569 |
| WBC, ×10^9^/L | 8.8±3.5 | 9.2±3.8 | 0.790 |
| Platelet, ×10^9^/L | 315.8±111.5 | 364.9±98.1 | 0.231 |
| ALT, U/L | 14.9±8.7 | 10.9±6.9 | 0.220 |
| Creatinine, μmol/L | 55.5(48.0-72.8) | 48.0(42.5-58.5) | 0.500 |
| BUN, μmol/L | 4.8±1.8 | 4.7±1.4 | 0.893 |
| **Inflammatory markers** |  |  |  |
| ESR, mm/h | 41.6±30.0 | 53.9±33.1 | 0.280 |
| CRP, g/L | 13.3(1.1-37.0) | 9.7(3.0-44.6) | 0.926 |
| C3, g/L | 1.14±0.26 | 1.37±0.27 | 0.033 |
| C4, g/L | 0.25±0.09 | 0.27±0.06 | 0.708 |
| CH50, g/L | 62.2±21.6 | 82.1±14.0 | 0.017 |
| IgG, g/L | 12.5±3.3 | 13.8±3.3 | 0.312 |
| IgA, g/L | 3.0(1.9-3.4) | 3.5(1.8-4.9) | 0.374 |
| IgE, g/L | 24.0(15.0-109.0) | 22.0(10.0-59.0) | 1.000 |
| IL-6, pg/ml | 9.3(3.9-19.2) | 10.1(3.7-18.8) | 0.710 |
| IL-8, pg/ml | 7.0(5.0-9.0) | 8.5(5.0-23.8) | 0.710 |
| SAA, mg/L | 22.2(5.1-183.5) | 77.9(11.9-219.0) | 1.000 |
| **immunosuppressants, n (%)** |  |  |  |
| 0 | 15(53.6) | 8(80.0) |  |
| 1 | 10(35.7) | 2(20.0) |  |
| 2 | 3(10.7) | 0 | 0.287 |
| **Treatment effect** |  |  |  |
| Complete remission | 17(60.7) | 3(30.0) | 0.144 |
| Partial remission | 7(25.0) | 4(40.0) | 0.432 |
| Relapse | 0 | 2(50.0) | 0.091 |

**Notes:** 1. Abbreviations: WBCs, white blood cells; ALT, alanine transaminase; Cr, creatinine; BUN, blood urine nitrogen; ESR, erythrocyte sedimentation rate; CRP, C-reactive protein; C3, complement 3; C4, complement 4; CH50, 50% hemolytic complement; IgG, immunoglobin G; IgA, immunoglobin A; IgE, immunoglobin E; IL-6, interleukin-6; IL-8, interleukin-8; SAA, serum amyloid A; NIH score, National Institutes of Health score; VISE, vascular ischemic symptoms and events.

**Supplementary Table S3**. The characteristics of patients with and without VISE among patients with VSP.

|  | **Non-VSP group** | | |  | **VSP group** | | |
| --- | --- | --- | --- | --- | --- | --- | --- |
|  | **Non-VISE group**  **(n = 18)** | **VISE group**  **(n = 2)** | ***p*-value** |  | **Non-VISE group**  **(n = 10)** | **VISE group**  **(n = 8)** | ***p*-value** |
| Age, years | 29.1±10.8 | 45.5±13.4 | 0.060 |  | 20.6±6.0 | 27.4±9.9 | 0.091 |
| Gender (Female), n (%) | 17(94.4) | 2(100.0) | 1.000 |  | 8(80.0) | 8(100) | 0.477 |
| Disease duration, months | 13.9(4.6-78.9) | 45.5(36.0-/) | 0.250 |  | 13.3(7.5-32.0) | 5.3(4.7-13.2) | 0.486 |
| VISE period, months | 11.0±5.6 | 19.9±12.5 | 0.070 |  | 6.3(5.6-7.9) | 8.0(4.9-10.5) | 0.637 |
| Naïve, n (%) | 8(44.4) | 0 | 0.495 |  | 4(40.0) | 6(75.0) | 0.188 |
| **Symptoms** |  |  |  |  |  |  |  |
| Fever, n (%) | 2(11.1) | 0 | 1.000 |  | 1(10.0) | 1(12.5) | 1.000 |
| Amaurosis, n (%) | 3(16.7) | 0 | 1.000 |  | 1(10.0) | 0 | 1.000 |
| Neck pain, n (%) | 3(16.7) | 0 | 1.000 |  | 1(10.0) | 1(12.5) | 1.000 |
| Visual loss, n (%) | 2(11.1) | 1(50.0) | 0.284 |  | 1(10.0) | 1(12.5) | 1.000 |
| Thoracalgia, n (%) | 2(11.1) | 1(50.0) | 0.284 |  | 2(20.0) | 1(12.5) | 1.000 |
| Fatigue, n (%) | 4(22.2) | 0 | 1.000 |  | 0 | 4(50.0) | 0.023 |
| **Signs** |  |  |  |  |  |  |  |
| Pulseless, n (%) | 4(22.2) | 1(50.0) | 0.447 |  | 2(22.2) | 4(50.0) | 0.335 |
| Vascular murmur, n (%) | 2(11.1) | 1(50.0) | 0.284 |  | 4(44.4) | 6(75.0) | 0.335 |
| **Laboratory parameters** |  |  |  |  |  |  |  |
| Hemoglobin, g/L | 110.7±24.0 | 123.5±0.7 | 0.472 |  | 111.4±13.9 | 113.9±11.9 | 0.706 |
| WBC, ×10^9^/L | 8.8±3.9 | 5.6±0.4 | 0.277 |  | 9.1± 2.4 | 9.9± 4.0 | 0.611 |
| Platelet, ×10^9^/L | 304.4±95.9 | 210.0±11.3 | 0.193 |  | 362.3±132.8 | 375.6±92.9 | 0.817 |
| ALT, U/L | 12.2±7.1 | 14.0±1.4 | 0.727 |  | 20.0(9.5-24.5) | 9.0(8.0-11.0) | 0.060 |
| Creatinine, μmol/L | 56.0(47.5-81.5) | 63.0(59.0-/) | 0.573 |  | 57.4±18.6 | 49.3±7.1 | 0.293 |
| BUN, μmol/L | 4.6±1.8 | 6.7±0.1 | 0.122 |  | 4.7(3.5-5.2) | 4.9(3.8-5.6) | 1.000 |
| **Inflammatory markers** |  |  |  |  |  |  |  |
| ESR, mm/h | 38.6 ±25.8 | 10.0 ±11.3 | 0.146 |  | 49.3±33.6 | 61.9±32.3 | 0.434 |
| CRP, g/L | 4.2(1.4-32.1) | 2.2(0.3-/) | 0.316 |  | 42.5±49.8 | 31.0±29.1 | 0.593 |
| C3, g/L | 1.09±0.17 | 0.94±0.35 | 0.275 |  | 1.27±0.32 | 1.43±0.28 | 0.325 |
| C4, g/L | 0.25(0.21-0.27) | 0.16(0.06-/) | 0.333 |  | 0.28±0.07 | 0.27±0.07 | 0.820 |
| CH50, g/L | 63.5±13.8 | 50.4±37.8 | 0.417 |  | 66.1±22.9 | 84.2±15.3 | 0.099 |
| IgG, g/L | 12.6±3.1 | 11.2±2.5 | 0.546 |  | 12.2±3.8 | 14.8±3.1 | 0.176 |
| IgA, g/L | 2.6±1.2 | 2.6±2.0 | 0.942 |  | 2.8(2.1-3.7) | 3.5(2.3-5.0) | 0.315 |
| IgE, g/L | 17.0(11.0-39.0) | 54.5(10.0-/) | 1.000 |  | 130.0(40.0-193.0) | 37.0(10.0-80.0) | 0.286 |
| IL-6, pg/ml | 9.1±7.4 | 25.5±30.8 | 0.050 |  | 17.8(5.9-27.5) | 13.4(4.5-25.3) | 0.637 |
| **immunosuppressants, n (%)** |  |  |  |  |  |  |  |
| 0 | 10(55.6) | 2(100.0) |  |  | 5(50.0) | 6(75.0) |  |
| 1 | 5(27.8) | 0 |  |  | 5(50.0) | 2(25.0) |  |
| 2 | 3(16.7) | 0 | 0.477 |  | 0 | 0 | 0.367 |

**Notes:** 1. Abbreviations: Hb, hemoglobin; WBCs, white blood cells; PLT, platelet; ALT, alanine transaminase; BUN, blood urine nitrogen; ESR, erythrocyte sedimentation rate; CRP, C-reactive protein; C3, complement 3; C4, complement 4; CH50, 50% hemolytic complement; IgG, immunoglobin G; IgA, immunoglobin A; IgE, immunoglobin E; IL-6, interleukin-6; VSP, vascular stenosis progression; VISE, vascular ischemic symptoms and events.

**Supplementary Table S4**. Factors associated with VISE in patients treated with tocilizumab.

|  | Univariate cox regression analysis | |  | Multivariate cox regression analysis | | |
| --- | --- | --- | --- | --- | --- | --- |
|  | HR (95% CI) | *p-*value |  | HR (95% CI) | *p*-value | |
| Age | 1.01(0.96-1.07) | 0.681 |  |  |  | |
| Gender | 0.04(0.00-333.2) | 0.488 |  |  |  | |
| Naïve | 1.92(0.54-6.86) | 0.313 |  |  |  | |
| Disease duration | 1.00(0.97-1.02) | 0.644 |  |  |  | |
| Hemoglobin | 1.00(0.97-1.04) | 0.799 |  |  |  | |
| Platelet | 1.01(1.00-1.01) | 0.074 |  |  |  | |
| ESR | 1.02(1.00-1.04) | 0.116 |  |  |  | |
| CRP | 1.00(0.98-1.02) | 0.890 |  |  |  | |
| IL-6 | 0.99(0.95-1.04) | 0.738 |  | 0.91(0.81-1.03) | 0.135 | |
| C3 | 14.17(1.39-143.94) | 0.025 |  | 254.45(2.19-29565.31) | 0.022 | |
| C3>1.0g/L | 0.03(0-9.77) | 0.229 |  |  |  |  |
| C4 | 2.72(0.00-2086.20) | 0.767 |  |  |  | |
| CH50 | 1.04(1.01-1.08) | 0.023 |  |  |  |  |
| NIH score | 1.36(0.61-3.04) | 0.449 |  |  |  | |

**Notes:** 1. Abbreviations: Hb, hemoglobin; PLT, platelet; G, globin; ESR, erythrocyte sedimentation rate; CRP, C-reactive protein; IL-6, interleukin-6; C3, complement 3; C4, complement 4; NIH Score, National Institutes of Health score; VISE, vascular ischemic symptoms and events. 2. All patients with VISE had high levels of C3 (≥1 g/L), and the results for different levels of C3 (C3 ≤ 1.0 g/L vs C3 > 1.0 g/L) were not significant.

**Supplementary Figure S1**. Manifestations of VISE in 10 patients as detected on MRA.

The blue words indicate VISE during the follow-up period, and the yellow arrow indicates the location of VSP.


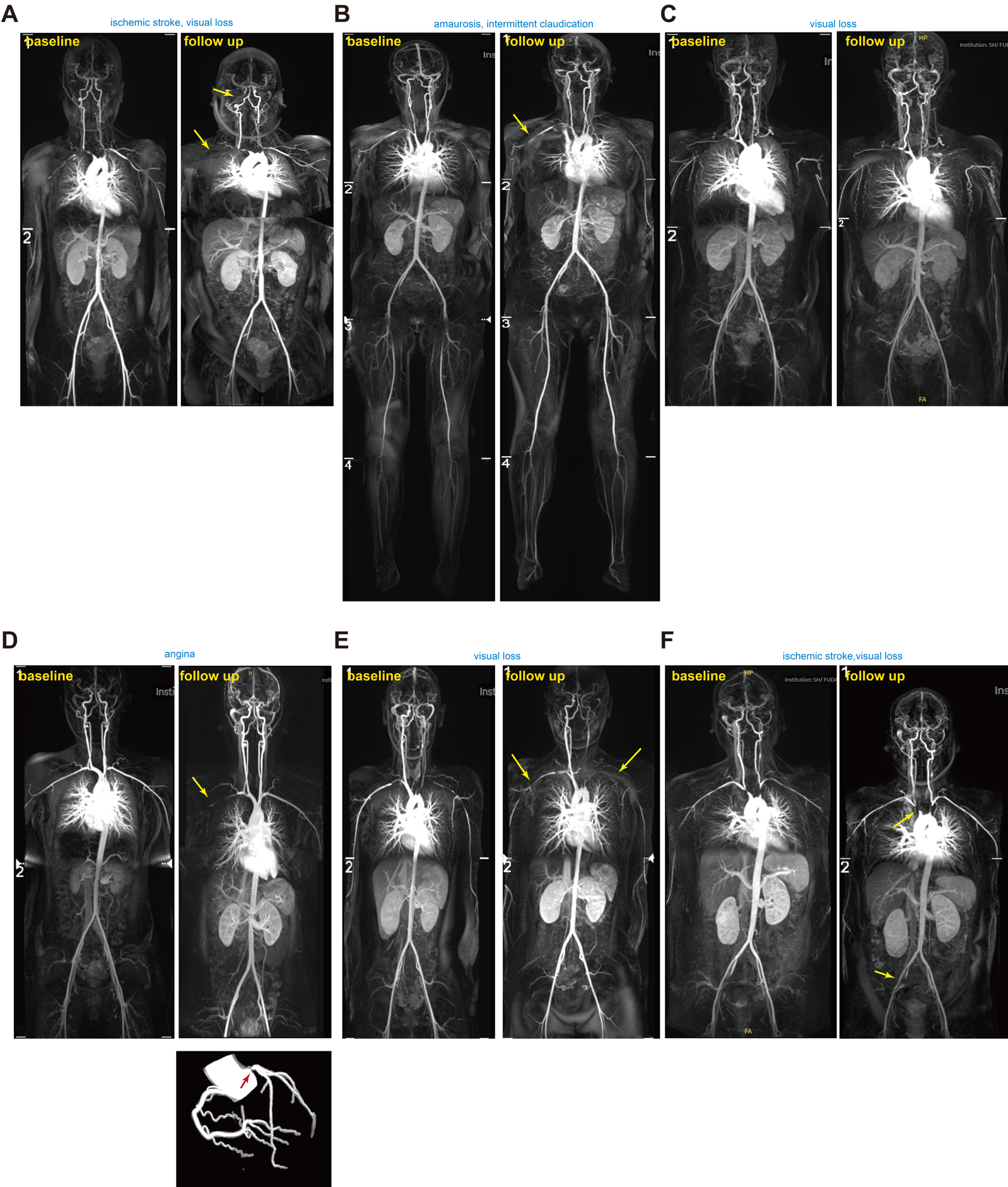


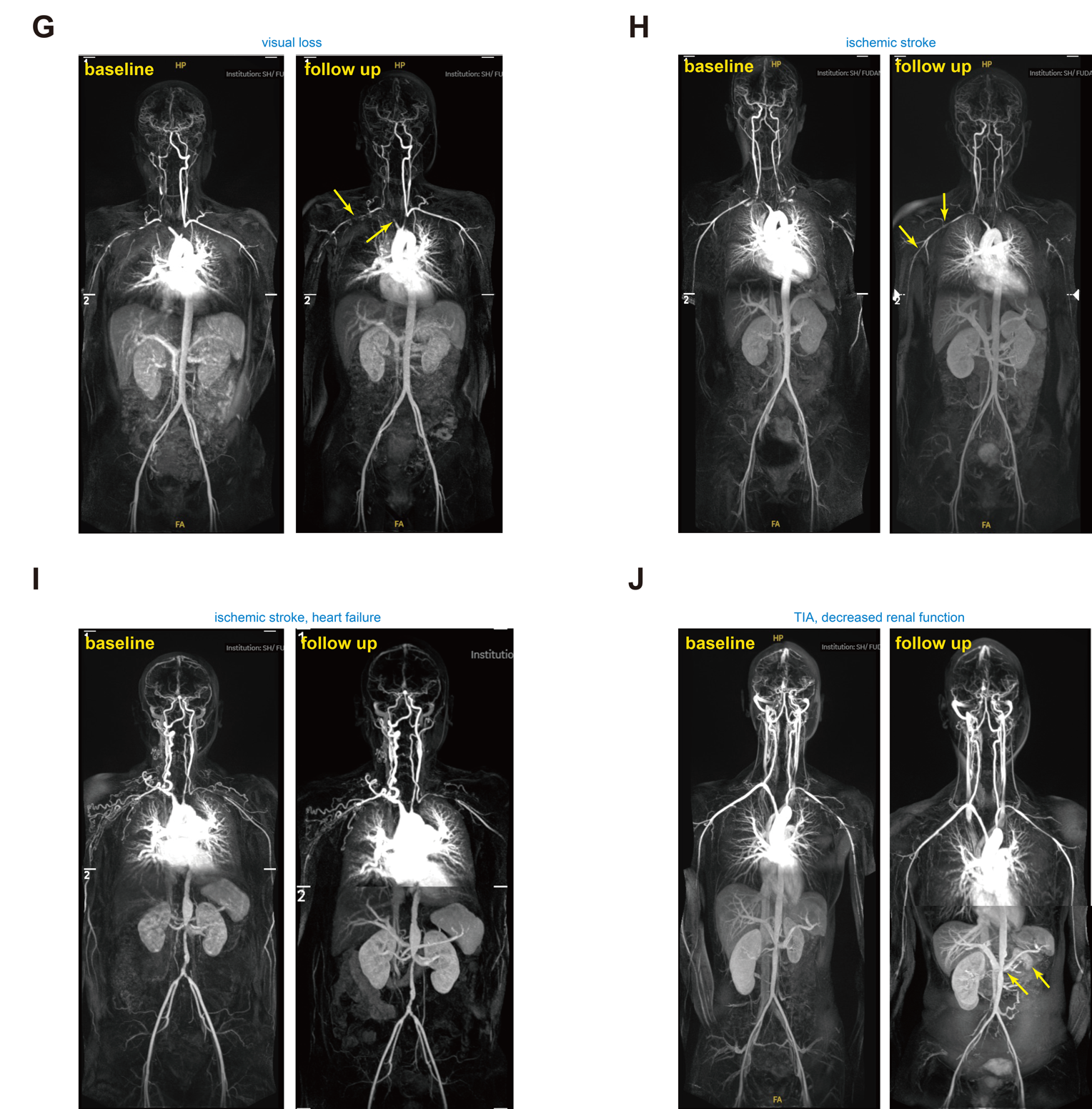


**Note**. The patient corresponding to figure D developed angina during the follow-up period, which was validated by CTA findings; however, evidence from baseline coronary CTA was lacking.

Abbreviations: VSP, vascular stenosis progression; VISE, vascular ischemic symptoms and events; MRA, magnetic resonance angiography; CTA, computed tomography angiography.

**Supplementary Figure S2**. Changes in inflammatory markers during the first 6 months of the follow-up period.

(A) The differences in inflammatory markers including ESR, CRP, C3, and IL-6 after treatment for 6 months in patients with and without VSP. (B) The difference in inflammatory markers including ESR, CRP, C3, and IL-6 after treatment for 6 months in patients with and without VISE. For example, ΔESR = ESR_baseline_ – ESR_6 months later_.

Abbreviations: VSP, vascular stenosis progression; VISE, vascular ischemic symptoms and events; ESR, erythrocyte sedimentation rate; CRP, C-reactive protein; complement 3; IL-6, interleukin-6.

**
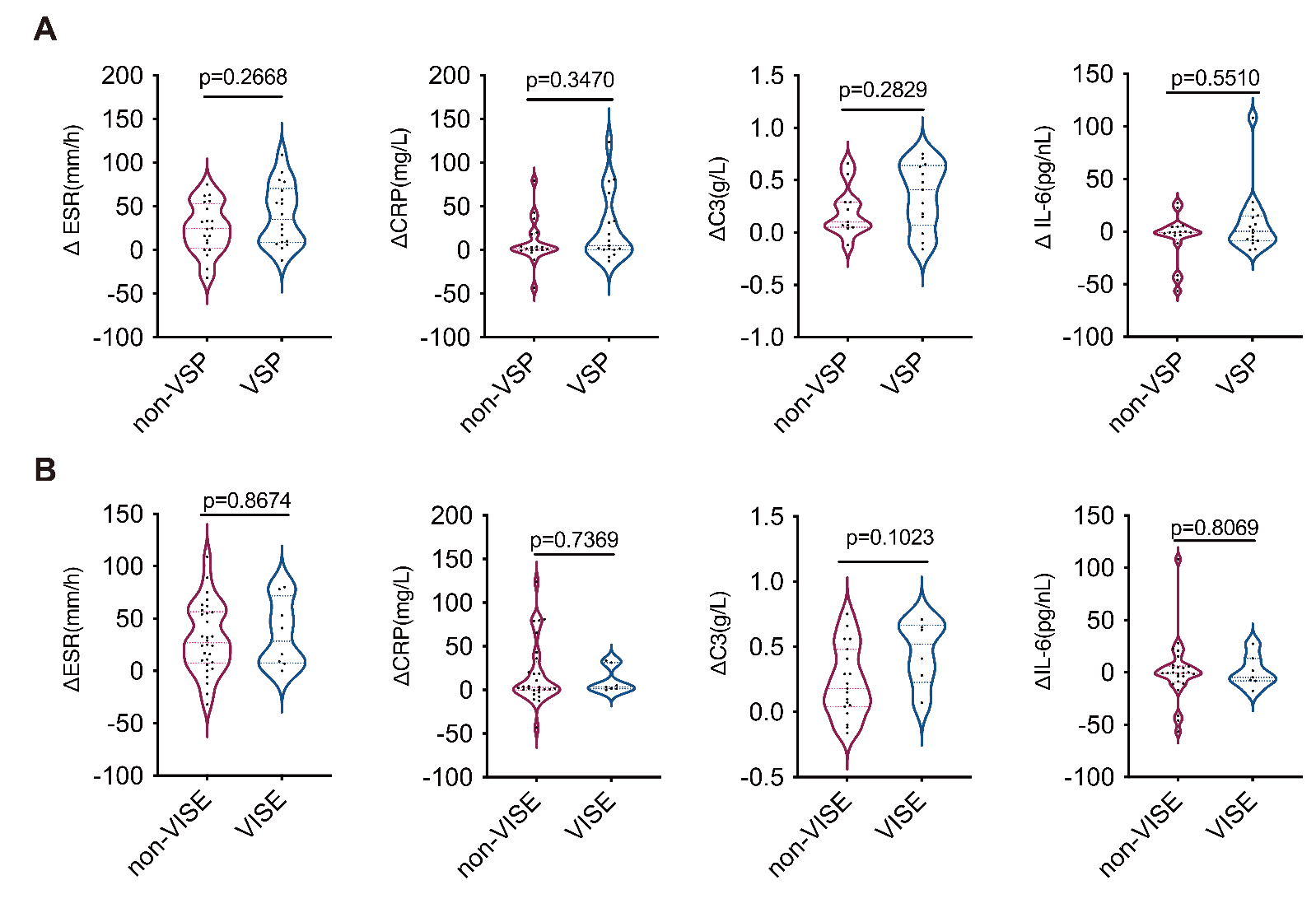
**

**Supplementary Figure S3**. The Kaplan-Meir curves of VSP and VISE for patients with different levels of NIH scores. (A) The Kaplan-Meir curve of VSP for patients with different NIH scores. (B) The Kaplan-Meir curve of VISE in patients with different levels of NIH scores.

Abbreviations: VSP, vascular stenosis progression; VISE, vascular ischemic symptoms and events.
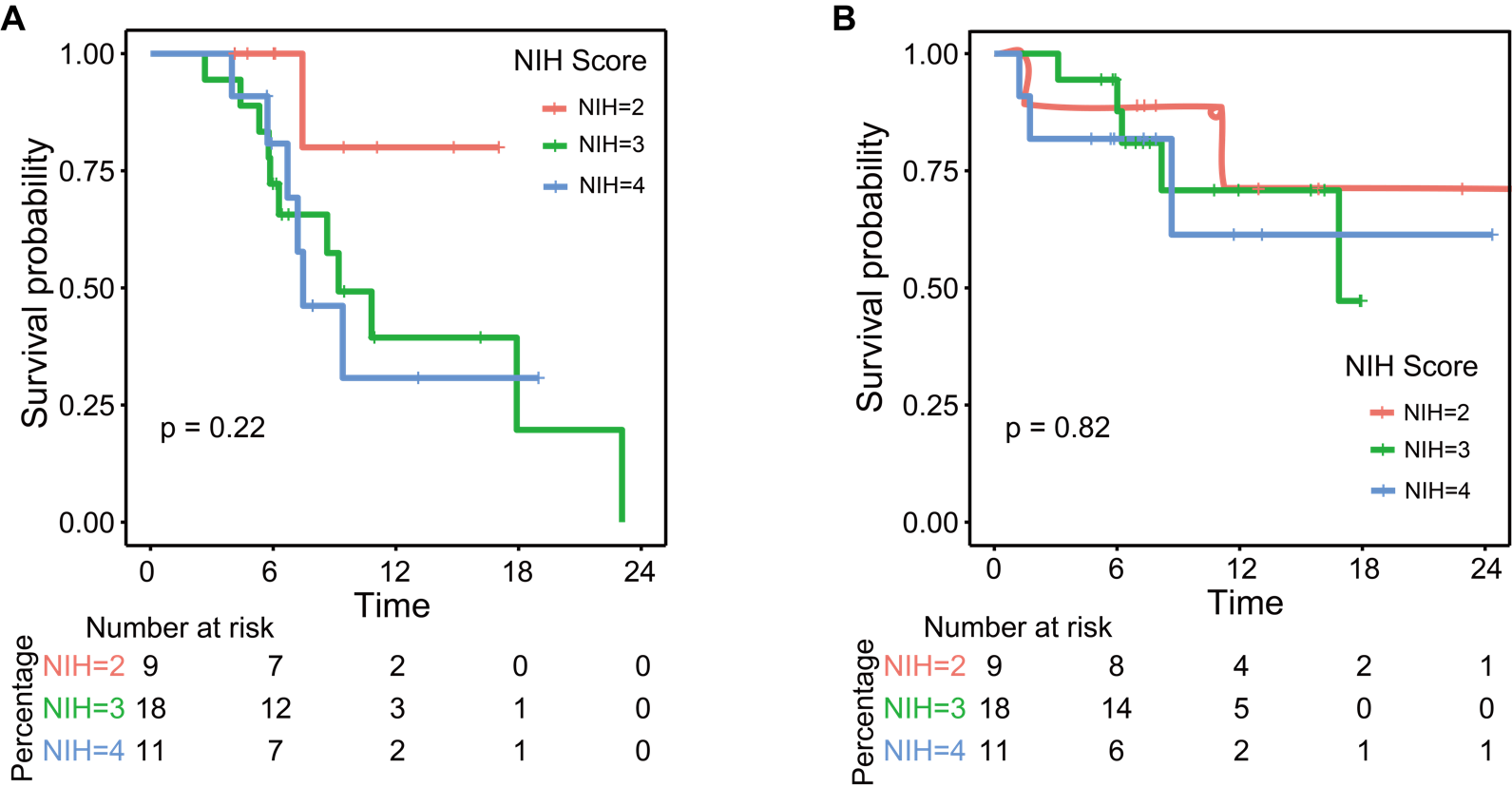

Supplement: Supplementary file 2 — Additional file 2: Supplementary Table S2-4 and Figure S1-3. Analysis of enrolled patients. [file 13075_2023_3106_MOESM2_ESM.docx]
